# LETTER TO THE EDITOR A Late Complication of Breast Augmentation With 2 Different Types of Injectable Materials

**Published:** 2011-04-20

**Authors:** Shunichi Nomoto, Rei Ogawa, Shigeyoshi Eura, Satoshi Hashimoto, Hiromi Kimura, Hiko Hyakusoku, Hiroshi Mizuno

**Affiliations:** Department of Plastic, Reconstructive and Aesthetic Surgery, Nippon Medical School, Tokyo, Japan

Dear Sir,

We experienced a very rare case of a severe complication after breast augmentation with 2 different types of injectable materials. At the ages of 22 and 35 years, a female who is now 74 years old underwent breast augmentation with injectable materials for cosmetic purposes at 2 different clinics. Ten years after the second augmentation procedures, breasts became itchy, and erythematous. These symptoms increased over time. At the patient's first visit to our hospital, both breasts exhibited erythema, subcutaneous varicoses, and deformities (Fig 1). On palpation, the laxity of the breast skin disappeared completely and the foreign body materials seemed to infiltrate into the subcutaneous tissue and breast parenchyma. Preoperative computed tomography (CT) showed diffuse radiopaque images in the superficial layer and solitary radiolucent images with eggshell-like calcification in the deep layer (Fig 1). Magnetic resonance imaging (MRI) generated both T1 high/T2 low and T1 low/T2 high images that were in accordance with the CT findings.

The patient underwent extirpation of the injected materials together with the affected skin, the breast parenchyma, and the pectoralis major muscles in the upper pole. This was followed immediately by reconstruction with rectus abdominis musculocutaneous flaps. Eight months later, the lower pole of the affected breast tissue and skin were removed along with the injected materials, followed immediately by reconstruction with latissimus dorsi musculocutaneous flaps. The patient was satisfied with both the symptomatic and cosmetic improvement. We also chemically analyzed the extracted substances by using ^13^C high-resolution magic angle spinning (MAS) nuclear magnetic resonance (NMR) spectroscopy. This analysis revealed clearly that there had been 2 different types of materials present in the breast: one type was in the superficial layer and was composed of hydrocarbon, and the other type was in the deep layer and consisted of silicone gel (Fig 2).

Injectable materials such as silicone gel and hydrocarbon compounds have been used for breast augmentation since the 1950s, particularly in Asian countries. Some patients who were augmented with these materials subsequently develop severe complications, including subcutaneous indurations, oily infiltration to the skin, calcification, and even systemic human adjuvant diseases.^1^^-^3 In the past, we have treated over 100 cases who complained of these complications by simply extirpating the materials and, in some cases, providing subsequent breast reconstructions by using autologous tissue transfer.^4^ However, none of the patients in our series had received more than 1 different injectable materials.

Previously, to identify the implant materials used for breast augmentation, we developed a clinical imaging technique using CT and MRI.^5^ If necessary, we also analyze the ingredients chemically by MAS NMR study. In the case reported here, where 2 different types of materials had been injected in the past, our preoperative diagnosis employing CT and MRI was completely consistent with the postoperative MAS NMR analysis of the injected materials and our experience with our series of breast-augmented patients. Thus, CT and MRI are highly reliable preoperative methods for determining the nature of the injected materials in breast-augmented patients.

## Figures and Tables

**Figure 1:**
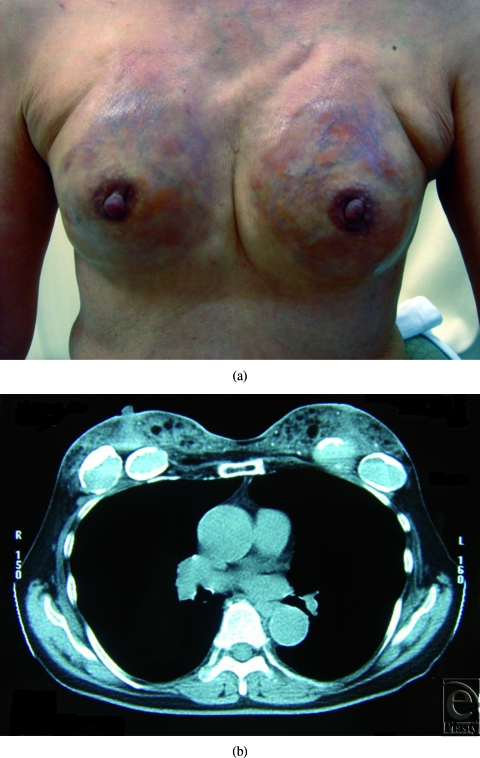
(*Left*) Preoperative appearance of the patient. (*Right*) Computed tomography revealed radiopaque images in the superficial layer and radiolucent images with eggshell-like calcification in the deep layer.

**Figure 2:**
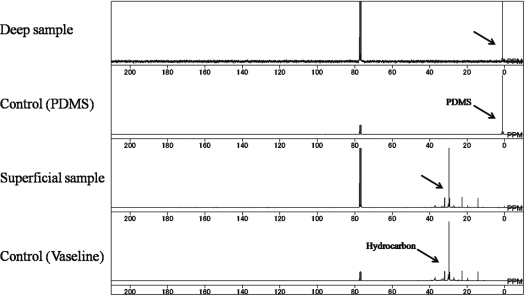
^13^C high-resolution magic angle spinning nuclear magnetic resonance revealed the presence in the extirpated matter of 2 different types of injectable materials, namely, silicone (same ingredient as polydimethyloxane or PDMS) and hydrocarbon.
